# Urine Metabolism Biomarkers Predict Preterm Infant Adiposity at Hospital Discharge

**DOI:** 10.1002/mnfr.70431

**Published:** 2026-03-12

**Authors:** Catherine O. Buck, Sarah McCollum, Weiwei Wang, TuKiet T. Lam, Sarah N. Taylor, Veronika Shabanova

**Affiliations:** ^1^ Department of Pediatrics Yale School of Medicine Connecticut USA; ^2^ Keck MS & Proteomics Resource Yale School of Medicine New Haven Connecticut USA; ^3^ Department of Molecular Biophysics and Biochemistry Yale University New Haven Connecticut USA

**Keywords:** body composition, diabetes in pregnancy, metabolomics, preterm, urine

## Abstract

In infants born to women with diabetes (DM), this study explores associations of urinary metabolite patterns with adiposity development in the newborn period. In term and preterm (30‐36 weeks gestational age) infants, body composition assessments were completed at hospital discharge. In urine samples from the first week, a targeted metabolomics assay was used. Quantile regression was used to evaluate associations of metabolite groups with DM in pregnancy and infant adiposity. Among 91 infants, 25 (27%) were exposed to DM, 68 (75%) were preterm. In the factor analysis, there was an association between factor 4 score (fatty acid and amino acid metabolites) and triceps skin fold thickness (β = 1.67 [95%CI: 0.62, 2.72]) and mid arm circumference (β = 1.59 [95%CI: 0.70, 2.49]) in preterm‐DM group. Urinary C2, C3, and ornithine were decreased in DM‐group (fold change <0.67, p < 0.01), and urinary C0, C2, C3, C5, ornithine, proline, and lysine were increased in preterm group (fold‐change >2.7, p<0.0001). In this cohort, infant urinary amino acid and acylcarnitine concentrations varied by DM during pregnancy and gestational age and were differentially related to postnatal adiposity in preterm DM‐group. Unique signatures of urinary metabolites may reflect early metabolism changes in the developing infant.

## Graphical abstract

In this cohort of term and preterm infants, urinary amino acid and acylcarnitine concentrations varied by diabetes in pregnancy exposure and gestational age at birth, and certain patterns of urinary metabolites were differentially related to postnatal adiposity development in preterm infants of women with diabetes in pregnancy.

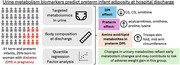

**Abbreviations**
DM: diabetes mellitus
FDR: false discovery rate
GA: gestational age
MUAC: mid upper‐arm circumference
SCFA: short chain fatty acids
SFT: skin fold thickness

## 1 Introduction

Evaluation of small molecular byproducts of physiologic pathways may identify related metabolism dyscrasias which impact the developmental programming for later health risks [1]. Perinatal exposures, such as obesity or diabetes during pregnancy/fetal development and preterm birth, relate to alterations in metabolite patterns in blood in newborns, and specific metabolites may be predictive of preterm birth‐related morbidities such as bronchopulmonary dysplasia or growth faltering [2, 3, 4, 5]. Urine is an alternative biological fluid which can be valuable in diagnostic assessments. Particularly in preterm infants, utilizing urine as a non‐invasive, convenient sampling method may provide easier integration of identified biomarkers into clinical decision making [6]. In newborns, urinary metabolites are associated with birthweight, gestational age and feeding practices [7, 8, 9, 10]. In preterm infants, the relationship of urinary metabolites with the antenatal environment during pregnancy and infant growth is largely unexplored.

Diabetes in pregnancy is associated with differences in early growth trajectory and greater adiposity in the newborn period among offspring, particularly in those born preterm [11, 12, 13]. In a prior study, our group determined unique patterns of serum metabolites related to protein and lipid metabolism among infants born preterm and in those born to women with diabetes in pregnancy [14]. These changes may reflect disturbances in nutrient metabolism and storage in this vulnerable group of infants with heightened risk of cardiometabolic disease [15, 16, 17]. To further characterize metabolism changes associated with perinatal exposures known to influence later growth and cardiometabolic outcomes, this study aims to compare patterns of urinary metabolites between term and preterm infants, and between infants born to women with and without diabetes in pregnancy, including how differences in urinary metabolites relate to adiposity development during the newborn hospitalization.

## 2 Methods

### 2.1 Study Population

Data for this study are from a prospective cohort of term and preterm infants admitted to the normal newborn nursery or level IV neonatal intensive care unit at Yale New Haven Hospital in New Haven, Connecticut, USA. The primary aim for this cohort was to examine the influence of diabetes in pregnancy on growth outcomes in moderate to late preterm infants. Therefore, infants born to women with diabetes in pregnancy were overenrolled during the recruitment process. Infants were enrolled prospectively between August 2020 and July 2022, and parents provided informed consent for their child to participate in the study. Preterm infants were born between 30 0/7 weeks and 36 6/7 weeks gestational age, and term infants were born at 37 0/7 weeks gestational age or greater. Exclusion criteria included parent age < 18 years, parental language not English or Spanish, and infant terminal illness, known genetic or chromosomal diagnoses, and/or congenital anomalies which may influence growth outcomes. Participants from the initial cohort with available urine samples for metabolomic analysis were included in this analysis. The study was approved by the Yale University Institutional Review Board and Clinical Study Registration Number was not required.

### 2.2 Body Composition Assessments

During the birth hospitalization, bedside anthropometry assessments were completed by a team of trained research nurses and coordinators. Assessments were completed at the time of study enrollment for both the term and preterm groups and repeated prior to hospital discharge for the preterm group if the length of hospital stay was anticipated to be more than 5 days. A length board was used to measure infant height using a standard two‐person method, and the measure recorded to the nearest 0.1 cm. Mid‐upper arm circumference (MUAC) was obtained using a paper measuring tape. Skin fold thicknesses (SFT) of the triceps and subscapular regions were obtained in duplicate using Lange skin fold calipers (Seko USA, Tullytown, PA) and previously described methods [18, 19, 20, 21]. Clinical measures of infant weight, head circumference, and abdominal circumference nearest to the time of the research measures was abstracted from the medical record.

### 2.3 Biological Sample Collection and Metabolomics Assays

In the first postnatal week, an infant urine sample was collected by placing an absorbent sponge in the diaper. The soaked sponge was then placed in a sterile container and stored between 2 and 8 °C until processing. Urine was extracted from the sponge by squeezing in a 30 mL empty syringe, and the sample was then aliquoted and stored at −80 °C until metabolomic analysis. Infant blood samples were obtained in the first two postnatal days, in conjunction with clinical lab draws done by trained hospital nurses. Cord blood samples were also obtained by draining blood from the umbilical cord after the placenta was delivered. Blood samples were stored at 2 to 8 °C until separation of serum, and subsequent storage at −80 °C. The earliest available blood sample for each infant was utilized in the metabolomic analysis.

A targeted, mass spectrometry‐based assay kit (AbsoluteIDQ p180, Biocrates Life Sciences AG, Innsbruck, Austria) was used to evaluate concentrations of 188 metabolites in each infants’ urine and blood sample. Urine and serum samples were prepared according to the kit manual were run on separate plates. Briefly, 10 µL of internal standard solution followed by 10 µL of infant sample were added to each well of the plate. The plates were then dried, and 50 µL of 5% phenyl‐isothiocyanate was added to derivatize the analytes. After drying again, metabolites were extracted with 300 µL of 5 mM ammonium acetate in methanol and subsequently diluted with 400 µL of running solvent. Flow injection analysis (FIA) was used to quantify sphingolipids, glycerophospholipids, acylcarnitines, and hexose. Tandem liquid chromatography/mass spectrometry (LC MS/MS) was used to quantify biogenic amines and amino acids using an Agilent High Pressure Liquid Chromatography System coupled to a 4000 Q‐Trap LC/MS/MS system. A Biocate kit precolumn and C18 analytical column (3.0 × 100 mm, 3.5 µm pore size) were also used. The p180 kit internal standards and quality control samples were used to assess the quality and robustness of the data and to calculate metabolite concentrations. The metabolomics analyses were conducted at the Keck MS and Proteomics Resource at Yale School of Medicine and Yale Center for Clinical Investigation. Mass spectral data was collected using Analyst software v1.7.2 (Sciex, Framingham, MA) and analyzed using Biocrates MetIDQ software version Oxygen‐DB110‐2976. Results of the serum metabolite analysis was previously published [14].

### 2.4 Covariates

Pregnancy and infant health characteristics were abstracted from the medical record by trained research staff. Pregnancy information included maternal race, ethnicity, delivery mode, diagnosis of diabetes mellitus (DM) in pregnancy (pre‐existing and/or gestational), and maternal obesity prior to pregnancy (body mass index > 30 kg/m^2^ either pre‐pregnancy or in the first trimester) [22, 23]. Infant health characteristics included infant sex, gestational age at birth, need for admission to the neonatal intensive care unit, and hospital length of stay. Diagnosis of respiratory distress during hospital stay was defined as respiratory distress syndrome, transient tachypnea, and/or retained lung fluid. Other infant health characteristics included hyperbilirubinemia, hypoglycemia (glucose < 40 mg/dL requiring intervention), use of intravenous fluids (such as dextrose containing fluids or parenteral nutrition) during the hospital stay, and any human milk at hospital discharge.

### 2.5 Statistical Analysis

In a bivariate analysis, infant and pregnancy outcomes were compared between the term and preterm groups. For the adiposity outcomes, gestational age‐ and sex‐specific weight, length, and head circumference z‐scores were calculated from Fenton reference curves [24]. The duplicate SFT measures were averaged, and percent body fat was calculated using a sex‐specific modified Slaughter method from the sum of the triceps and subscapular SFT measurements [25, 26, 27]. For the analyses associating urine metabolism markers with adiposity outcomes, estimates from birth were utilized for term infants, and estimates at hospital discharge were utilized for preterm infants. This is so that adiposity outcomes were compared at a similar developmental timepoint point for both groups, which is near term‐age equivalent.

A total of 79 metabolites from the initial 188 included in the targeted assay were excluded from the metabolite analysis due to missing data in more than 20% of subjects (Table S1) [28]. This was either due to the metabolite either not being detected or the concentration being below the level of detection for the analysis system. The remaining 109 metabolites were included in (1) a factor analysis to identify groups of correlated metabolites and (2) an individual metabolite analysis.

To examine individual compounds associated with DM in pregnancy and prematurity, we compared the fold change (ratio of concentrations) in each of the metabolites measured using the student's t‐test. We then plotted the fold change against the log_10_(*p*‐value) in a volcano plot for the comparisons of the DM group versus the non‐DM group and the term group versus the preterm group. QIAGEN IPA (QIAGEN Inc., https://digitalinsights.qiagen.com/IPA, Redwood City, CA) was used to identify pathway‐level associations of individual metabolites with prematurity, DM in pregnancy, and adiposity outcomes of interest [29]. Individual metabolites were first identified using the following publicly available databases: bioBDnet [30], MBROLE 2.0 [31], PubChem [32], and HMDB [33]. All 109 metabolites identified in the urine samples were identified within the IPA database. Log‐fold change in the concentrations of each metabolite were compared between the four groups according to DM in pregnancy and GA at birth, and metabolites were examined according to known pathways, biological diseases, and functions in the IPA database. In the analyses of individual metabolites, a two‐sided alpha of 0.05 was used in the hypothesis tests, with the Benjamini–Hochberg approach applied to control the False Discovery Rate (FDR) [34]. We also assessed the relationship between blood and urine metabolite concentrations with a Spearman's rank correlation coefficient. We then used the Benjamini–Hochberg procedure to assess significant correlations in each of the 6 classes of compounds assessed by the metabolomics assay.

For the exploratory factor analysis, the number of factors was plotted against the eigenvalues in a Scree plot, and the final number of factors (four) was chosen by checking the total variance explained by subsequent factors and based on an eigenvalue > 1. We excluded individual metabolites (N = 8, Table S2) which had factor loading < 0.7 and/or were loaded onto more than one factor from the factor analysis [35, 36]. A factor score for each infant was outputted, and scores were compared between the term and preterm groups and between the DM and non‐DM groups using the Wilcoxon rank sum test. We then used quantile regressions to explore the change in the median of each adiposity outcome per unit change in factor score, stratified by both diabetes in pregnancy and gestational age. The width and coverage of 95% confidence intervals (95% CI) for the estimated slopes were used to evaluate effect sizes and clinically meaningful differences in the relationship between the factor scores and discharge adiposity. For example, meaningful differences were discussed, if a 95% CI covered the effect sizes in mostly one direction and did not cross the value of 0 in the opposite direction by a magnitude reflective of a clinically or physiologically relevant magnitude. In order not to overfit our regression models due to the sample size within combinations of diabetes in pregnancy and gestational age, we approached confounding on statistical grounds where we a priori specified that only covariates with meaningful differences by diabetes in pregnancy and gestational age in the bivariate analysis would be considered for inclusion in the final regression models. None of the observed covariates reached that, therefore, the quantile regression models only included the main effects of diabetes, gestational age at birth (term versus preterm) and their statistical interaction, with the latter facilitating estimation of slopes in stratified analyses. SAS version 9.4 (Cary, NC) and R statistical software were used for the study analyses.

## 3 Results

Among 150 infants in the initial cohort, 91 with available urine metabolomics data were included in this analysis. A total of 25/91 (27%) infants were born to women with DM in pregnancy, and 68/91 (75%) infants were born preterm, with average gestational age (GA) of 34.4 weeks. Infants in the preterm group had higher average length of stay (13 days vs 4 days, p<0.001), smaller birth weight z‐score (−0.9 vs 0.1, p = 0.002) and smaller mid upper arm circumference (9.0 mm vs 10.0 mm, p<0.001) at hospital discharge, compared with term infants (Table 1). Other pregnancy and infant health characteristics were similar between the term and preterm group.

**TABLE 1:** Pregnancy and infant characteristics according to gestational age at birth (N = 91).

	Preterm group^a^ (N = 68)	Term group (N = 23)	*p*‐value^*^
Pregnancy characteristics			
Race			0.43
Black	9 (13)	5 (22)	
White	53 (78)	15 (65)	
Other	6 (9)	3 (13)	
Hispanic ethnicity	15 (22)	7 (30)	0.42
Diabetes^b^	20 (29)	5 (22)	0.48
Pre‐pregnancy BMI (kg/m^b^)	28.1 (24.0, 36.2)	29.3 (25.2, 36.3)	0.70
C‐section delivery	42 (62)	10 (43)	0.13
Multiple gestation	24 (35)	0 (0)	<0.001
Infant characteristics			
Female sex	31 (46)	10 (43)	0.86
Gestational age at birth (weeks)	34.4 (33.6, 35.2)	39.0 (37.9, 39.7)	<0.001
NICU admission	66 (97)	20 (87)	0.10
Length of stay (days)	13.0 (8.0, 28.0)	4.0 (3.0, 5.0)	<0.001
Hypoglycemia	29 (43)	12 (52)	0.43
Respiratory distress	39 (57)	12 (52)	0.67
Hyperbilirubinemia	16 (24)	02 (9)	0.14
Any IV fluids^c^	40 (59)	09 (39)	0.10
Human milk at discharge	49 (72)	20 (87)	0.15
Term‐equivalent age anthropometry			
Weight z‐score	−0.9 (−1.5, −0.2)	0.1 (−0.9 – 0.7)	0.002
Mid upper arm circumference (mm)	9.0 (7.8, 9.8)	10.0 (9.8, 11.5)	<0.001
Triceps skin fold thickness (mm)	3.9 (3.0, 5.3)	4.4 (3.8, 5.0)	0.18
Subscapular skin fold thickness (mm)	3.8 (3.0, 4.5)	4.0 (3.3, 5.0)	0.26
Estimated body fat percentage^d^	7.1 (5.0, 9.4)	8.5 (6.1, 9.5)	0.14
Postmenstrual age at measure (weeks)	35.6 (35.0, 36.5)	39.0 (37.9, 39.7)	<0.001

Data present as N (%) or Median (25^th^, 75^th^ percentile).

Abbreviation: BMI (body mass index), NICU (neonatal intensive care), IV (intravenous).

^a^ Preterm group: 30 0/7 to 36 6/7 gestational age.

^b^ Type 1, type 2, or gestational diabetes.

^c^ Receipt of any intravenous fluids or parenteral nutrition during hospital stay.

^d^ Calculated using modified Slaughter method (Slaughter 1988; Aris 2013).

* Wilcoxon rank sum test for continuous variables, Chi‐square or Fisher's Exact test for categorical variables.

### 3.1 Individual Metabolites

**FIGURE 1:**
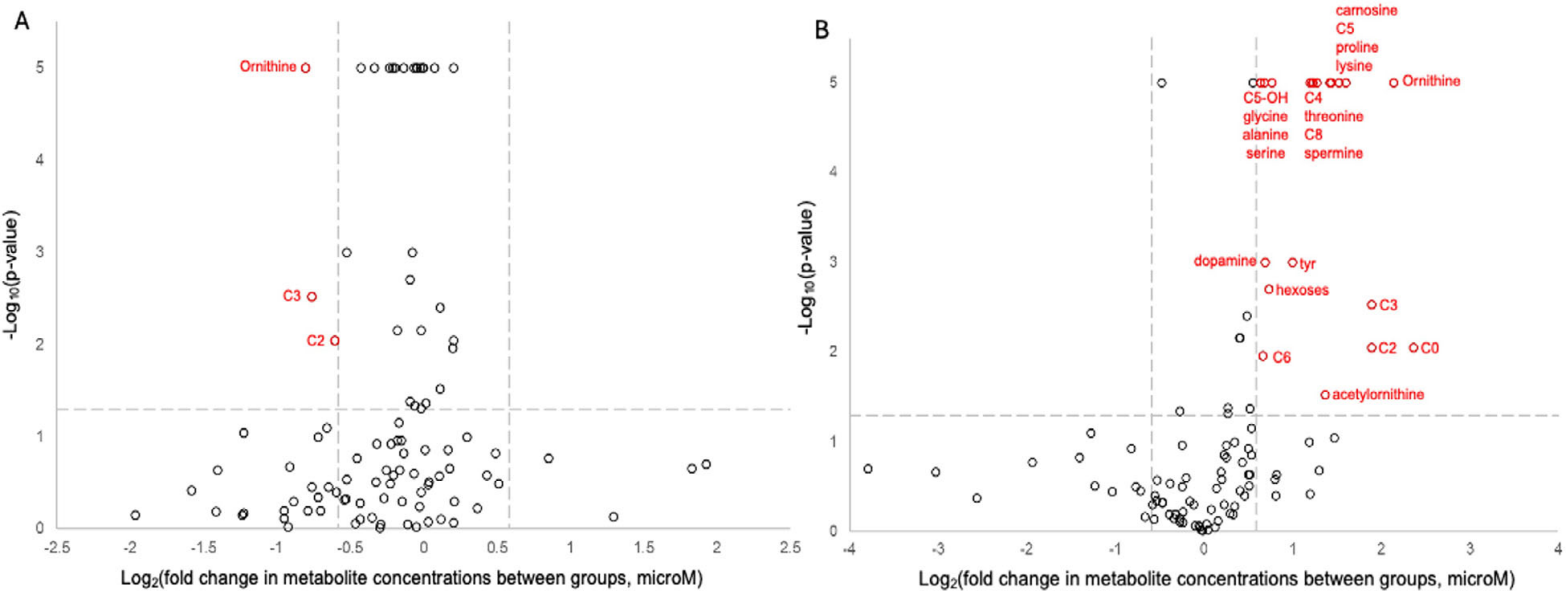
Volcano plot of log‐fold change in urine metabolite concentrations by (A) diabetes group vs non‐diabetes group and (B) preterm group vs term group. Metabolites with log‐fold change > 1.5 and t‐test p‐value < 0.05 (adjusted for False Discovery Rate) are labeled and indicated in red on the plots.

In the analysis of individual metabolites, there was differential expression of metabolites according gestational age groups and DM groups (Figure 1). Urinary concentrations of C2 (0.66‐fold change, p = 0.009), C3 (0.59‐fold change, p = 0.003), and ornithine (0.57 fold change, p<0.0001) were decreased in the DM‐group relative to the non‐DM group. Compared to the term group, preterm infants had an increased expression of urinary acylcarnitines C0 (fold change 5.14, p = 0.009), C2 (fold change 3.71, p = 0.009), C3 (fold change 3.71, p = 0.003), C4 (fold change 2.43, p<0.0001), C5 (fold change 2.88, p<0.0001), C5‐OH (fold change 1.71, p<0.0001), C6 (fold change 1.58, p = 0.011), and C8 (fold change 2.33, p<0.0001). Additionally, urinary concentrations of the amino acids alanine, glycine, lysine, ornithine, proline, serine, and threonine were between 1.56 and 4.42 fold change higher in preterm infants compared with term infants (p<0.0001). Urinary carnosine and hexose concentrations were also 3.03 (p<0.0001) and 1.66 (p = 0.002) fold higher in preterm infants compared with term infants, respectively.

**FIGURE 2:**
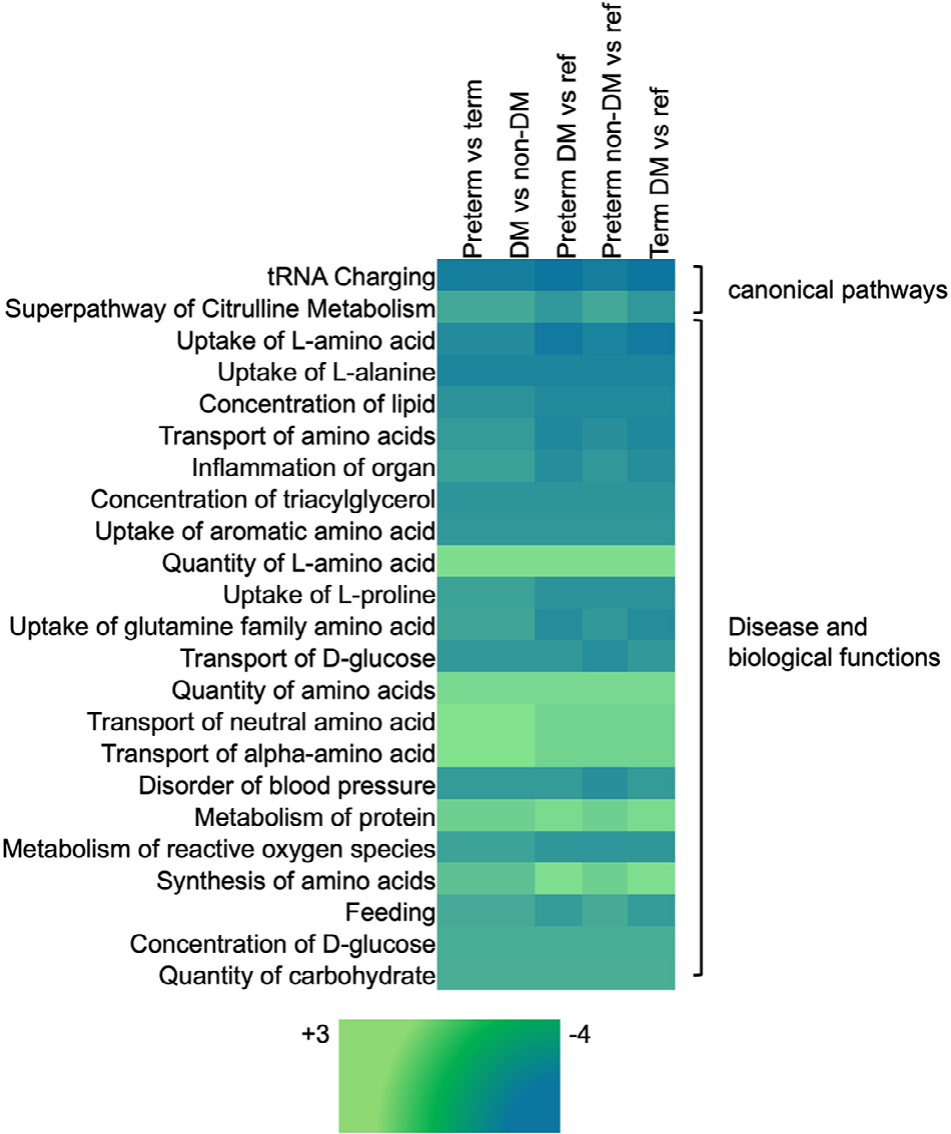
Heat map of activation z‐scores for biological functions and pathways associated with infant urine metabolites, according to gestational age and diabetes (DM) in pregnancy group, with the reference (ref) group as the term, non‐DM group.

In the individual pathway analysis of urinary metabolites, there were meaningful differences across gestational age and diabetes in pregnancy groups in pathways related to synthesis, uptake, and transport of amino acids, protein metabolism, lipid metabolism, and the super pathway of citrulline metabolism (Figure 2). Among the 91 infants included in the initial analysis, 75 (82%) had available metabolite data for both blood samples and urinary samples from the first postnatal week. In those infants, there was a moderate correlation between infant blood and infant urine concentrations of histidine, isoleucine, leucine, valine, and hexose in the first postnatal week (Table 2; Spearman's correlation coefficient: 0.40–0.46, p<0.001). There was also a weaker correlation between blood and urinary concentrations of acylcarnitines C16:1 and C2, amino acids threonine, phenylalanine and arginine (p < 0.01).

**TABLE 2:** Correlation of urine and serum metabolites (N = 75 subjects).

Compound class	Metabolite name	Correlation coefficient^1^	*p*‐value^2^
Acylcarnitines	C16:1	0.32	0.005
	C2	0.30	0.009
Amino acids	Leu	0.47	0.0001
	Ile	0.41	0.0003
	Val	0.41	0.0003
	His	0.40	0.001
	Thr	0.39	0.001
	Phe	0.38	0.001
	Arg	0.31	0.01
	Tyr	0.29	0.01
	Met	0.26	0.03
	Trp	0.24	0.04
Biogenic amines	alpha‐AAA	0.34	0.003
Glycerophospholipids	PC aa C40:6	0.35	0.002
Sugars	H1	0.46	<0.0001

^1^ Spearman's rank correlation coefficient.

^2^ *p*‐value from Benjamini‐Hochberg correction to control for False Discovery Rate.

### 3.2 Factor Analysis

In the exploratory factor analysis, four different factors of metabolite groupings were identified (Table S3). Factor 1 included several amino acids, glycerophospholipds, and sphingolipids, as well as 3 specific biogenic amines (dopamine, acetylornithine, and kynurenine). Factor 2 contained the sphingolipid SM C22:3 and the majority of the glycerophospholipids. Factor 3 contained several short chain acylcarnitines, the glycerophospholipid PC aa C38:0, and the biogenic amines dopamine and putrescine. Lastly, factor 4 included hexose, the glycerophospholipid lysoPC a C16:0, the biogenic amines carnosine, asymmetric dimethylarginine and spermine, and several amino acids including glycine, ornithine, lysine, and arginine. There were meaningful differences between the term and preterm groups in factor 4 score, with the preterm group having higher median factor 4 scores compared with the term group (Table 3). Scores from factors 1, 2 and 3 were not meaningfully different between gestational age groups. Factor scores were not different between the DM and non‐DM groups (Table S4).

**TABLE 3:** Median factor scores from exploratory factor analysis of urine metabolites by infant gestational age.

	Preterm infants (N = 62)	Term infants (N = 29)	*p*‐value^*^
Factor 1 (23% of variability)	−0.2 (‐0.3, ‐0.1)	−0.2 (−0.3, ‐0.1)	0.60
Factor 2 (18% of variability)	−0.2 (‐0.3, ‐0.0)	−0.2 (−0.3, 0.1)	0.54
Factor 3 (12% of variability)	−0.3 (‐0.5, 0.1)	−0.3 (−0.5, 0.0)	0.81
Factor 4 (9% of variability)	0.3 (‐0.2, 0.9)	−0.8 (−1.1, ‐0.7)	<0.001

Data presented as median (Q1, Q3).

* Wilcoxon rank sum test.

**FIGURE 3:**
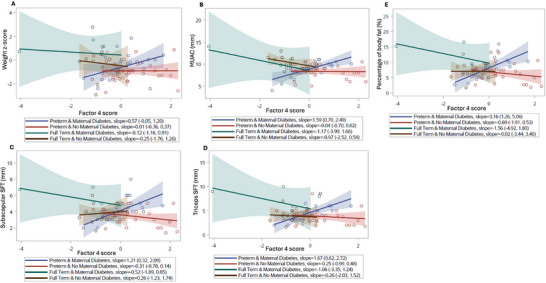
Unadjusted quantile regression estimates of (A) weight z‐score, (B) mid‐upper arm circumference (MUAC), (C) subscapular skin fold thickness (SFT), (D) triceps SFT and (E) estimated body fat percentage at term age according to Factor 4 scores. Results are grouped by combinations of gestational age (full term or preterm) and diabetes (DM) in pregnancy. Slopes represent 𝞫 value from quantile regression analysis (change in median of outcome for every one unit change in Factor 4), with surrounding 95% confidence intervals.

The analysis of the association of metabolite factor scores with adiposity outcomes near the time of hospital discharge revealed differences in the direction of the association between factor 4 score and infant adiposity when comparing DM and gestational age groups (Figure 3). In the preterm DM group, factor 4 scores were positively associated with near discharge mid arm circumference [estimated slope 1.59 (95% CI: 0.70, 2.49)], triceps SFT [estimated slope 1.67 (95% CI: 0.62, 2.72)], subscapular SFT [estimated slope 1.21 (95% CI: 0.32, 2.09)], and estimated percent body fat [estimated slope 3.16 (95% CI: 1.26, 5.06)].

## 4 Discussion

In this cohort study of term and preterm infants, patterns of urinary metabolites were associated differentially with gestational age at birth, the perinatal environment of diabetes during pregnancy, and adiposity development after preterm birth. Urinary concentrations of short‐chain acylcarnitines, sugar, and several amino acids were higher in preterm infants, with groupings of some of these metabolites associated with higher measures of total and subcutaneous body fat in the group born to women with diabetes in pregnancy. Together, these findings highlight potential metabolic signatures that are reflective of differences in the way energy and nutrients are utilized in this group. As future studies link these metabolism biomarkers with longitudinal infant growth and metabolic outcomes, integration of urinary metabolites into the nutritional care of preterm infants may allow for additional tailoring of nutritional management in this group at high‐risk for altered early growth.

To our knowledge, this study is the first to directly relate alterations in non‐invasive urinary markers in preterm infants with assessments of adiposity development in the newborn period. In a pilot study of 20 infants, Morniroli et al. compared body composition in the first 3 months of age and concentrations of urinary metabolites using an untargeted approach between term and preterm infants [37]. In that study, preterm infants had higher concentrations of urinary choline/phosphocholine, betaine, and glucose compared with term infants. Additionally, preterm infants had higher fat mass as term equivalent age, however, that study did not directly relate the urinary metabolite findings with the body composition findings in the statistical analysis. A different study of 90 term infants examined cross‐sectional associations of urinary metabolites with weight and length in infants under age four months, finding that choline and some byproducts of the tricarboxylic acid cycle were positively associated with infant weight [38]. In the present study, we found that concentrations of carnosine and lysine, which were concentrated in Factor 4 from the factor analysis, were positively associated with peripheral fat measures in preterm DM‐group infants. This is contrary to the findings in the study described above, in which lysine and carnosine were inversely correlated with infant weight [38]. The difference in findings between the present study and prior studies is likely related to heterogeneity in the gestational ages of infants and chronologic ages in the infants in each of the cohorts.

Urinary metabolomic profiling in newborns is a valuable, non‐invasive approach to understanding early life metabolic processes and their potential links to later health outcomes, including obesity and cardiometabolic diseases. In preterm infants with growth restriction, one study showed elevated concentrations of myo‐inositol, sarcosine, creatine and creatinine, patterns which are linked to cardiovascular disease risk in other studies [39]. These patterns may reflect developmental re‐programing, as early alterations in urinary metabolites among those born preterm may persist into adulthood [40]. In adults, elevated concentrations of both branched‐chain amino acids and acylcarnitines are linked to insulin resistance, central adiposity, and increased cardiovascular risk [41]. Elevation in these specific metabolites may reflect underlying mitochondrial dysfunction and altered nutrient handling [42]. For example, acylcarnitines are generated when fatty acid oxidation processes in the mitochondria are overloaded and/or the beta‐oxidation process is incomplete, alterations which are linked with insulin resistance and lipid accumulation in adults [43, 44]. Elevations in excreted and circulating branched chain amino acids may reflect altered transamination of these amino acids, which may in turn may alter the way cellular metabolism and nutrient sensing pathways (e.g. mTOR) are activated [45, 46].

In the present study, we found a significant correlation of urinary and serum metabolites, including acylcarnitines and several branched chain amino acids. We have previously demonstrated that alterations in these metabolites in serum in preterm infants relate to both diabetes in pregnancy and adiposity outcomes at hospital discharge [14]. Strong correlation of urinary and serum metabolites indicates that urine may provide a practical and non‐invasive tool for biomarker‐based risk stratification in neonatal nutritional care. With further validation, urinary metabolite patterns could complement existing growth and clinical assessments to identify infants at risk for altered growth and metabolic health.

Mechanistically, the alterations in urinary metabolites across gestational age and diabetes in pregnancy groups in this study may also reflect differences in the nutrition that the groups received. Our between‐group analysis did not reveal meaningful differences in the nutritional supplementation between groups. This includes formula versus human milk intake and need for intravenous fluids and/or parenteral nutrition during the newborn hospitalization. However, prior studies have found that nutritional intakes do relate to urinary metabolites in both term and preterm infants. For example, there are shifts in the upregulation of pathways related to protein and lipid metabolism among very preterm infants born prior to 29 weeks gestational age receiving full enteral feeds compared with those receiving parenteral nutrition [8].

Previous studies have also demonstrated changes over time (days to months) in urinary metabolite profiles in term infants, which may relate to dynamic changes in dietary intakes in early infancy [47, 48]. In the present study, preterm infants had higher urinary amino acids and acylcarnitines compared with term infants, which could relate to protein intake in the preterm group. In moderate to late preterm infants, it is common practice to supplement human milk feeds with additional calories and/or to use a post‐discharge formula which is higher in protein than standard term formulas [49, 50]. In adults, protein intake relates directly with urinary concentrations of amino acids [51, 52]. Additionally, studies of term infants have found distinct patterns of urinary metabolites among infants feeding human milk compared with those feeding infant formulas with varying protein contents [53, 54].

It is possible that alterations in urinary metabolites reflect changes in the composition and function of the gut microbiome, which are otherwise linked with the development of obesity and cardiometabolic disease [55, 56]. In one of the previously mentioned studies of term infants, alterations in some urinary metabolites correlate with stool metabolites related to protein metabolism, including short chain fatty acids (SCFA) and tyrosine and phenylalanine [53]. In newborns, fecal short chain fatty acids are produced by anaerobic fermentation of intestinal carbohydrates and protein by gut bacteria, and the proportion of specific SCFA's depend on the enrichment of different families of gut microbes [56, 57]. While the present study does not include an analysis of stool metabolites or microbiome components, alterations in urinary metabolites have related directly to the gut microbiome in another research [58]. During pregnancy, for example, specific taxa of gut microbes are associated with urinary markers of insulin resistance [59]. Emerging evidence demonstrates that some components of the microbiome and related metabolome in women with metabolic conditions in pregnancy are transmitted to the newborn [60, 61, 62]. Additional studies are required to understand the complex relationship between the perinatal environment, such as metabolic health conditions in pregnancy, newborn nutritional exposures (including human milk), and markers of infant metabolism. To this effect, our finding that urinary spermine (and its precursor ornithine) concentrations were higher in preterm infants and correlated with growth outcomes in the preterm diabetes‐exposed group of infants may relate to human milk intake. Spermine is a polyamine which is present in higher concentrations in human milk compared with formula, is an important regulator of growth and gastrointestinal tract maturation [63, 64]. Animal models have demonstrated that spermine supplementation relates to an increase in bifidobacteria colonization of the gut, a microbiome profile which is inversely related to obesity [65].

Strengths of our study include moderate sample size of prospective cohort of term and preterm infants. Our cohort also includes robust data regarding both urinary and serum metabolites in the newborn period, and body composition assessments beyond standard anthropometry of weight and length. Together, these data provide important information regarding how the perinatal environment influences growth and metabolism in the newborn period.

Limitations of this study include the inability to include data regarding control of diabetes during pregnancy and detailed nutritional exposure information for the infant into the statistical analysis, limited ability to test for sex effects, and lack of longitudinal follow‐up data. While women with diabetes in pregnancy are at risk of preterm birth, it is not feasible to enroll a preterm cohort of infants prenatally. Therefore, we were limited to available medical record data to phenotype the metabolic health of the mothers of infants in this study. Lack of data regarding diabetes control, such as hemoglobin A1C values or glucose concentrations during pregnancy may have introduced bias into the analysis presented in this study. Additionally, we did not find differences in the nutritional intakes and infant sex between diabetes in pregnancy groups in our sample, which may rule out these variables as confounders on empirical grounds, and given our sample size we did not include these variables in our statistical regression models. We are therefore unable to assess how differences in some potential confounders, such as infant sex, infant medication exposures, and early nutritional exposures may have independently influenced the observed variation in urinary metabolites in this study. Larger cohort studies with longitudinal sample collection and follow‐up data are also required to further tease out the lasting effects of the associations revealed in this cohort study. Lastly, our methodology included a targeted metabolomic assay, which limits our ability to detect a broad range of metabolites. In our study, 79/188 metabolites were below the level of detection or not detected in our subjects. While this proportion is in line with the detection of metabolites using similar targeted metabolomics approaches in newborns [66, 67], we are unable to assess how low concentrations of some of these metabolites may have impacted growth in our population.

In summary, in this cohort study we found that urinary amino acid and acylcarnitine concentrations varied by diabetes in pregnancy and gestational age, and that certain patterns of urinary metabolites were differentially related to postnatal adiposity development in preterm infants born to women with diabetes in pregnancy. These unique signatures of urinary metabolites may reflect early metabolism changes, including alterations in fatty acid oxidation and amino acid metabolism in the developing infant, and contribute to subsequent risk of adverse weight gain and metabolic health in this group of infants. These results highlight the potential for urinary metabolite profiling to serve not only as a research tool but also as a clinically applicable, non‐invasive approach to monitor infants at risk for the development of derangements in metabolic health.

## Funding

This study was, in part, made possible by NIH Grant Number K23HD104907; NIH/CTSA Grant Number UL1TR001863 from NCATS; the COVID‐19 fund to retain clinical scientists at Yale, sponsored by the Doris Duke Charitable Foundation award #2021266, and the Yale Center for Clinical Investigation; the Robert E. Leet and Clara Guthrie Patterson Trust Mentored Research Award, Bank of America, Private Bank, Trustee; and the Society for Pediatric Research bridging to success award. The 4000 QTRAP mass spectrometer at the Keck MS & Proteomics was funded by NIH/CTSA UL1RR024139 from NCATS.

## Conflicts of Interest

The authors declare no conflicts of interest.

## Supporting information




**Supporting File**: mnfr70431‐sup‐0001‐SupMat.docx.
