# Supplementary material for: Urine Metabolism Biomarkers Predict Preterm Infant Adiposity at Hospital Discharge
Source: Mol Nutr Food Res. 2026 Mar 12;70(6):e70431. doi: 10.1002/mnfr.70431 (PMC12981208; doi:10.1002/mnfr.70431)
Supplement: Supplementary file 1 — Supporting File: mnfr70431‐sup‐0001‐SupMat.docx. [file MNFR-70-e70431-s001.docx]

Buck et al.

Urine metabolism biomarkers and preterm infant adiposity

**Supplemental Tables and Figures**

**Supplemental Table 1**: Urine metabolites not detected during analysis (N = 79 metabolites)

| **Compound class** | **Compound name** | | | |
| --- | --- | --- | --- | --- |
| Acylcarnitines | Butyrylcarnitine  Hexenoylcarnitine  Dodecanoylcarnitine  Dodecenoylcarnitine  Tetradecenoylcarnitine | Hydroxytetradecenoylcarnitine  Tetradecadienylcarnitine  Hydroxyhexadecanoylcarnitine  Hydroxyhexadecenoylcarnitine  Hexadecadienylcarnitine | | Hydroxyhexadecadienylcarnitine  Octadecanoylcarnitine  Octadecenoylcarnitine  Hydroxyoctadecenoylcarnitine  Octadecadienylcarnitine |
| Aminoacids | Glutamate | | | |
| Biogenic Amines | Carnosine  Dihydroxyphenylalanine  *cis*-4-Hydroxyproline  *trans*-4-Hydroxyproline  Methionine sulfoxide | Nitrotyrosine  Serotonin  Spermidine  Symmetric dimethylarginine | | |
| Glycerophospholipids | lysoPhosphatidylcholine acyl C17:0  lysoPhosphatidylcholine acyl C18:0  lysoPhosphatidylcholine acyl C18:1  lysoPhosphatidylcholine acyl C18:2  lysoPhosphatidylcholine acyl C20:3  lysoPhosphatidylcholine acyl C24:0  lysoPhosphatidylcholine acyl C28:0  lysoPhosphatidylcholine acyl C28:1  Phosphatidylcholine diacyl C24:0  Phosphatidylcholine diacyl C28:1  Phosphatidylcholine diacyl C30:2  Phosphatidylcholine diacyl C32:1  Phosphatidylcholine diacyl C32:3  Phosphatidylcholine diacyl C34:3  Phosphatidylcholine diacyl C34:4  Phosphatidylcholine diacyl C36:0  Phosphatidylcholine diacyl C36:6  Phosphatidylcholine diacyl C38:1  Phosphatidylcholine diacyl C38:3  Phosphatidylcholine diacyl C40:1  Phosphatidylcholine diacyl C40:2  Phosphatidylcholine diacyl C40:3  Phosphatidylcholine diacyl C42:0  Phosphatidylcholine diacyl C42:1  Phosphatidylcholine diacyl C42:2 | | Phosphatidylcholine diacyl C42:4  Phosphatidylcholine diacyl C42:5  Phosphatidylcholine diacyl C42:6  Phosphatidylcholine acyl-alkyl C30:0  Phosphatidylcholine acyl-alkyl C32:2  Phosphatidylcholine acyl-alkyl C34:1  Phosphatidylcholine acyl-alkyl C34:2  Phosphatidylcholine acyl-alkyl C36:0  Phosphatidylcholine acyl-alkyl C36:4  Phosphatidylcholine acyl-alkyl C38:0  Phosphatidylcholine acyl-alkyl C38:1  Phosphatidylcholine acyl-alkyl C38:2  Phosphatidylcholine acyl-alkyl C38:3  Phosphatidylcholine acyl-alkyl C38:5  Phosphatidylcholine acyl-alkyl C38:6  Phosphatidylcholine acyl-alkyl C40:1  Phosphatidylcholine acyl-alkyl C40:3  Phosphatidylcholine acyl-alkyl C40:4  Phosphatidylcholine acyl-alkyl C42:0  Phosphatidylcholine acyl-alkyl C42:1  Phosphatidylcholine acyl-alkyl C42:5  Phosphatidylcholine acyl-alkyl C44:3  Phosphatidylcholine acyl-alkyl C44:4  Phosphatidylcholine acyl-alkyl C44:5  Phosphatidylcholine acyl-alkyl C44:6 | |
| Sphingolipids | Sphingomyeline C20:2  Hydroxysphingomyeline C22:1  Hydroxysphingomyeline C22:2  Sphingomyeline C26:0 | | | |

**Supplemental table 2**: Urine metabolites excluded from factor analysis due to factor loading in >1 factor (N=8)

| **Compound class** | **Compound name** |
| --- | --- |
| Acylcarnitines | Propenoylcarnitine  Hexadecanoylcarnitine |
| Biogenic Amines | alpha-Aminoadipic acid  Sarcosine  Taurine |
| Glycerophospholipids | lysoPhosphatidylcholine acyl C16:1  Phosphatidylcholine diacyl C26:0  Phosphatidylcholine diacyl C32:0 |

**Supplemental table 3:** Factor group loadings from exploratory factor analysis of urine metabolites (N=89 metababolites)

| **Factor** | **Compound class** | **Compound name** | | |
| --- | --- | --- | --- | --- |
| 1 | Acylcarnitines | C14:2-OH | | |
| 1 | Amino acids | Asn  Asp  Glu  Ile | Leu  Phe  Trp  Val | |
| 1 | Biogenic Amines | Ac-Orn  Dopamine  Met-SO | | |
| 1 | Glycerosphingolipids | lysoPC a C14:0  PC aa C30:0  PC aa C36:1 | PC ae C34:0  PC ae C36:1 | |
| 1 | Sphingolipids | SM (OH) C14:1  SM (OH) C16:1  SM (OH) C22:1  SM (OH) C22:2  SM (OH) C24:1 | SM C16:0  SM C18:0  SM C24:0  SM C24:1  SM C26:1 | |
| 2 | Glycerosphingolipids | lysoPC a C20:4  lysoPC a C26:0  lysoPC a C26:1  PC aa C32:2  PC aa C34:2  PC aa C36:2  PC aa C36:3  PC aa C36:4  PC aa C36:5 | PC aa C38:4  PC aa C38:5  PC aa C38:6  PC aa C40:4  PC aa C40:5  PC aa C40:6  PC ae C30:1  PC ae C30:2  PC ae C32:1 | PC ae C34:3  PC ae C36:2  PC ae C36:5  PC ae C38:4  PC ae C40:2  PC ae C40:5  PC ae C42:2  PC ae C42:3  PC ae C42:4 |
| 2 | Sphingolipid | SM C22:3 | | |
| 3 | Acylcarnitines | C0  C2  C3  C3-DC (C4-OH)  C4:1  C5-DC (C6-OH) | C5-M-DC  C5:1  C5:1-DC  C6 (C4:1-DC)  C7-DC  C9 | |
| 3 | Biogenic amines | DOPA  Putrescine | | |
| 3 | Glycerophospholipids | PC aa C38:0 | | |
| 4 | Acylcarnitines | C5  C5-OH (C3-DC-M)  C8 | C12-DC  C14  C16:1 | |
| 4 | Amino acids | Arg  Cit  Gly  His | Lys  Orn  Ser  Tyr | |
| 4 | Biogenic amines | alpha-AAA  Carnosine  t4-OH-Pro | | |
| 4 | Glycerophospholipds | lysoPC a C16:0 | | |
| 4 | Sugar (hexose) | H1 | | |

**Supplemental table 4:** Mean factor scores for exploratory factor analysis of urine metabolites by exposure to diabetes in pregnancy

|  | **Non-DM group**  **(N = 64)** | **DM group**  **(N = 27)** | **P value*** |
| --- | --- | --- | --- |
| Factor 1 (23% of variability) | 0.07 (-0.48 – 8.43) | -0.09 (-0.60 – 1.13) | NS |
| Factor 2 (18% of variability) | 0.07 (-0.71 – 8.57) | -0.09 (-0.49 – 1.02) | NS |
| Factor 3 (12% of variability) | -0.17 (-0.97 – 1.35) | 0.03 (-0.77 – 4.63) | NS |
| Factor 4 (9% of variability) | 0.06 (-1.66 – 2.28) | -0.14 (-4.06 – 1.70) | NS |

Data presented as mean (range)

*Wilcoxon rank sum test
